# Simulated Gastric Acid Promotes the Horizontal Transfer of Multidrug Resistance Genes across Bacteria in the Gastrointestinal Tract at Elevated pH Levels

**DOI:** 10.1128/spectrum.04820-22

**Published:** 2023-04-18

**Authors:** Hai-yan Wu, Zi-lin Wei, Dan-yang Shi, Hai-bei Li, Xin-mei Li, Dong Yang, Shu-qing Zhou, Xue-xia Peng, Zhong-wei Yang, Jing Yin, Tian-jiao Chen, Jun-wen Li, Min Jin

**Affiliations:** a Department of Environment and Health, Tianjin Institute of Environmental and Operational Medicine, Key Laboratory of Risk Assessment and Control for Environment and Food Safety, Tianjin, China; USDA-ARS

**Keywords:** simulated gastric fluid, pH, antibiotic-resistant bacteria, horizontal gene transfer, genome-wide RNA sequencing

## Abstract

The assessment of factors that can promote the transmission of antibiotic resistance genes (ARGs) across bacteria in the gastrointestinal tract is in great demand to understand the occurrence of infections related to antibiotic-resistant bacteria (ARB) in humans. However, whether acid-resistant enteric bacteria can promote ARG transmission in gastric fluid under high-pH conditions remains unknown. This study assessed the effects of simulated gastric fluid (SGF) at different pH levels on the RP4 plasmid-mediated conjugative transfer of ARGs. Moreover, transcriptomic analysis, measurement of reactive oxygen species (ROS) levels, assessment of cell membrane permeability, and real-time quantitative assessment of the expression of key genes were performed to identify the underlying mechanisms. The frequency of conjugative transfer was the highest in SGF at pH 4.5. Antidepressant consumption and certain dietary factors further negatively impacted this situation, with 5.66-fold and 4.26-fold increases in the conjugative transfer frequency being noted upon the addition of sertraline and 10% glucose, respectively, compared with that in the control group without any additives. The induction of ROS generation, the activation of cellular antioxidant systems, increases in cell membrane permeability, and the promotion of adhesive pilus formation were factors potentially contributing to the increased transfer frequency. These findings indicate that conjugative transfer could be enhanced under certain circumstances in SGF at elevated pH levels, thereby facilitating ARG transmission in the gastrointestinal tract.

**IMPORTANCE** The low pH of gastric acid kills unwanted microorganisms, in turn affecting their inhabitation in the intestine. Hence, studies on the factors that influence antibiotic resistance gene (ARG) propagation in the gastrointestinal tract and on the underlying mechanisms are limited. In this study, we constructed a conjugative transfer model in the presence of simulated gastric fluid (SGF) and found that SGF could promote the dissemination of ARGs under high-pH conditions. Furthermore, antidepressant consumption and certain dietary factors could negatively impact this situation. Transcriptomic analysis and a reactive oxygen species assay revealed the overproduction of reactive oxygen species as a potential mechanism by which SGF could promote conjugative transfer. This finding can help provide a comprehensive understanding of the bloom of antibiotic-resistant bacteria in the body and create awareness regarding the risk of ARG transmission due to certain diseases or an improper diet and the subsequent decrease in gastric acid levels.

## INTRODUCTION

Antibiotic resistance has been considered a public health threat that prevents the treatment of bacterial infections and results in a continual increase in mortality (1). Hundreds to thousands of deaths are reported annually because of infections related to antibiotic-resistant bacteria (ARB) (2). At least 2.8 million ARB-related infections occur in the United States every year, with 35,000 deaths being attributed to such infections (3). As the gastrointestinal tract is transiently inhabited by pathogens in diseased states, resistance elements in the gut may be transferred to clinically relevant pathogens. Therefore, the transmission of antibiotic resistance genes (ARGs) in the gastrointestinal tract has become a research hot spot to assess ARB-related infections in humans.

Bacteria acquire antibiotic resistance mainly through vertical gene transfer (VGT) and horizontal gene transfer (HGT), including conjugation, transformation, and transduction (4, 5). ARG transmission in the gastrointestinal tract may be attributed to the long-term consumption of antibiotic or nonantibiotic pharmaceuticals or residual exposure to these from food and drinking water (6), which can result in VGT or HGT in the gastrointestinal tract. However, the identification of other factors that can promote an ARB bloom in the gastrointestinal tract is a topic of great research interest.

In general, gastric fluid in the stomach is the central component of the digestive process (7). The low pH of gastric fluid plays an important role as an ecological filter to kill unwanted microbial taxa that would otherwise colonize the intestines (8). However, the intestinal pathogens Escherichia coli and Shigella flexneri are more acid resistant than other bacteria and therefore can survive after exposure to pH 2.0 ± 2.5. Moreover, Salmonella enterica serovar Typhimurium cells are killed following exposure to pH 3.0 (9). The intestinal pathogen count may reach 10^9^ in contaminated food stored for 1 to 2 days at 20°C (10), and the consumption of such food may lead to the HGT of acid-resistant bacteria in humans because the ingested food can stay in the stomach for 3 to 5 h. Previous studies have demonstrated that the chlorination process, which kills most bacteria in water, can promote the horizontal transfer of plasmids by natural transformation and conjugative transfer, resulting in the exchange of ARGs across bacterial genera and the transfer of chlorine-resistant opportunistic pathogens from non-ARB to ARB (11, 12). Helicobacter pylori, a Gram-negative bacterium that colonizes the human stomach, is naturally competent for transformation. Some studies have revealed that the horizontal transfer of genes in H. pylori can occur via plasmid-mediated transformation (13, 14). However, whether acid-resistant enteric bacteria can enhance the horizontal transfer of ARGs in gastric fluid under high-pH conditions remains unclear.

In this study, simulated gastric fluid (SGF) was prepared *in vitro* to assess whether it could facilitate the transmission of ARGs within and across bacterial genera through conjugative transfer. Furthermore, the effects of pH levels, the times of exposure to SGF, drug consumption, and dietary factors on the conjugative transfer of ARGs were assessed. In order to unravel the mechanism underlying the enhancement of conjugative transfer, transcriptome resequencing, measurement of reactive oxygen species (ROS) levels, assessment of cell membrane permeability, and reverse transcription-quantitative PCR (RT-qPCR) analysis were performed to explore key channels. To the best of our knowledge, this is the first study to reveal the enhancement of conjugative transfer under certain circumstances in SGF, which can facilitate the transmission of ARGs in the gastrointestinal tract. Our findings can help provide a comprehensive understanding of ARB blooms in the body.

## RESULTS

### Effects of the pH levels of SGF on the conjugative transfer of ARGs.

**FIG 1:**
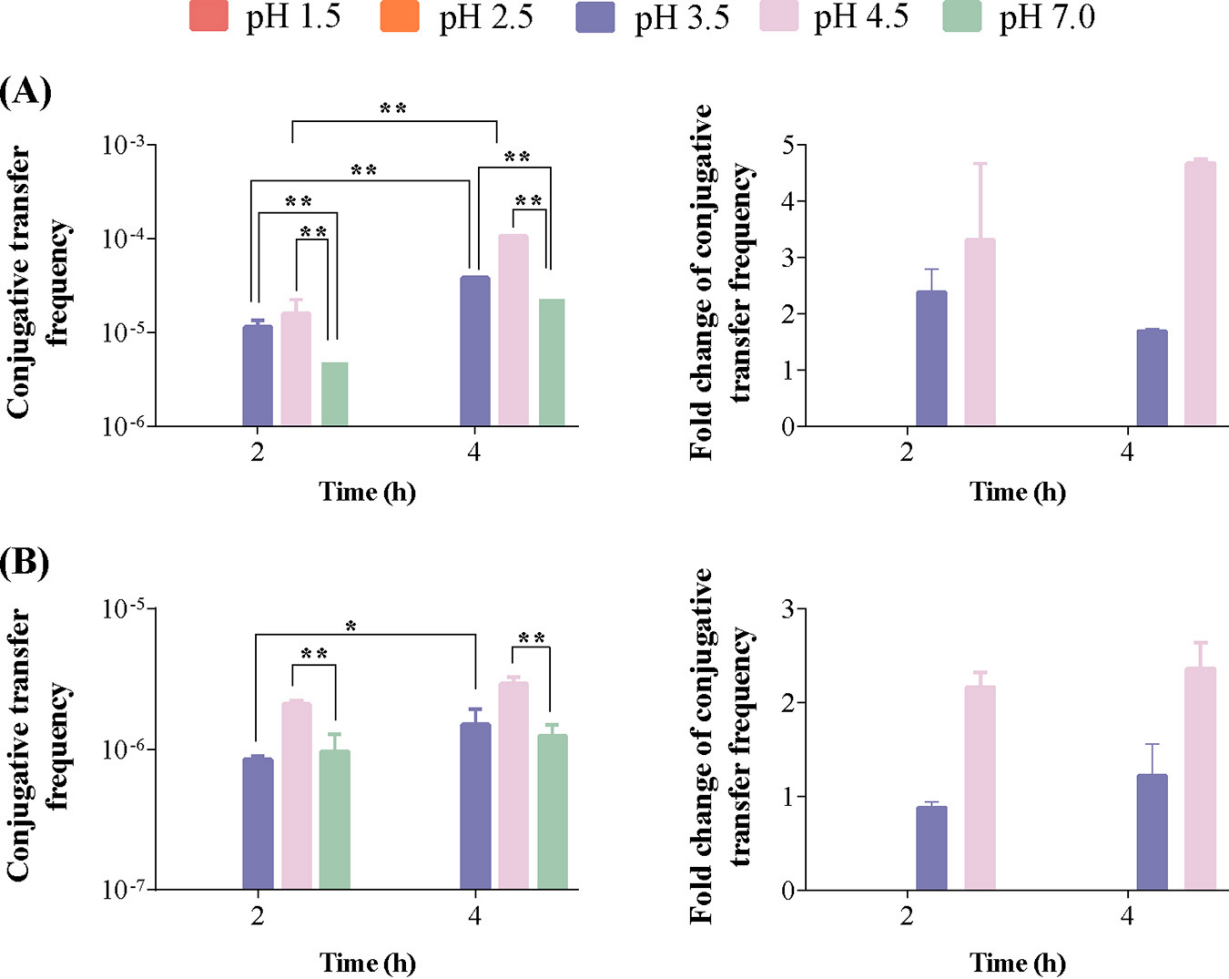
Effects of SGF pH and exposure time on the frequency of conjugative transfer (left) and fold changes in the frequency of conjugative transfer (right) of the RP4 plasmid from donor E. coli HB101 cells to recipient E. coli K-12 cells (A) and Salmonella Aberdeen cells (B). The bars denote means ± standard deviations (SDs) from three separate experiments. Significant differences are shown with * (*P < *0.05) and ** (*P < *0.01).

To assess the effects of SGF at different pH levels on the horizontal transfer of ARGs within and across bacterial genera, the conjugative transfer of the RP4 plasmid exposed to SGF at various pH levels (1.5, 2.5, 3.5, or 4.5) or phosphate-buffered saline (PBS) (pH 7.0) was performed with an exposure time of 4 h. As shown in Fig. 1, no transconjugants were noted on the plates exposed to SGF at pH 1.5 and 2.5. However, when E. coli HB101 and E. coli K-12 were exposed to SGF together at pH 3.5 and 4.5, transconjugants began to grow on the plates, and the conjugative transfer frequencies reached (3.79 ± 0.11) × 10^−5^ and (1.05 ± 0.17) × 10^−4^, 1.68-fold and 4.66-fold higher than that in the control group at pH 7.0, respectively (*P < *0.01). In contrast, when E. coli and Salmonella enterica serovar Aberdeen were exposed to SGF together at pH 3.5, no evident difference was noted between the conjugative frequencies at pH 3.5 and pH 7.0 (control). Moreover, when E. coli and Salmonella Aberdeen were exposed to SGF together at pH 4.5, the conjugative transfer frequency was significantly increased to (2.91 ± 0.34) × 10^−6^, 2.36-fold higher than that in the control group at pH 7.0 (*P < *0.01). This may be due to the higher rate of mortality of the recipient Salmonella Aberdeen cells at pH 3.5 (64.54%) than that of the recipient E. coli K-12 cells at pH 3.5 (38.92%). On the other hand, no significant difference was noted between the mortality of the recipient Salmonella Aberdeen cells (14.38%) and that of the recipient E. coli K-12 cells (17.56%) at pH 4.5 (see Fig. S2 in the supplemental material). Thus, SGF can significantly promote the horizontal transfer of ARGs within and across bacterial genera when its pH is maintained at 3.5 or 4.5.

### Effects of the time of exposure to SGF on the conjugative transfer of ARGs.

To determine the effects of the time of exposure to SGF on the horizontal transfer of ARGs within and across bacterial genera, the conjugative transfer of the RP4 plasmid was performed after exposure to SGF for various times. As shown in Fig. 1, significant differences were noted in the conjugative transfer frequencies between E. coli HB101 and E. coli K-12 at exposure times of 2 and 4 h when the pH was 3.5. The conjugative transfer frequency at 4 h was 3.34-fold higher than that in the control group at 2 h (*P < *0.01). In addition, when the pH was 3.5, the frequency of conjugative transfer between E. coli and Salmonella Aberdeen was significantly increased to (1.50 ± 0.43) × 10^−6^ at 4 h, 1.75-fold higher than that in the control group at 2 h (*P < *0.05). Thus, the horizontal transfer of ARGs across bacteria may be improved by increasing the time of exposure to gastric fluid.

### Effects of drug consumption on the conjugative transfer of ARGs.

**FIG 2:**
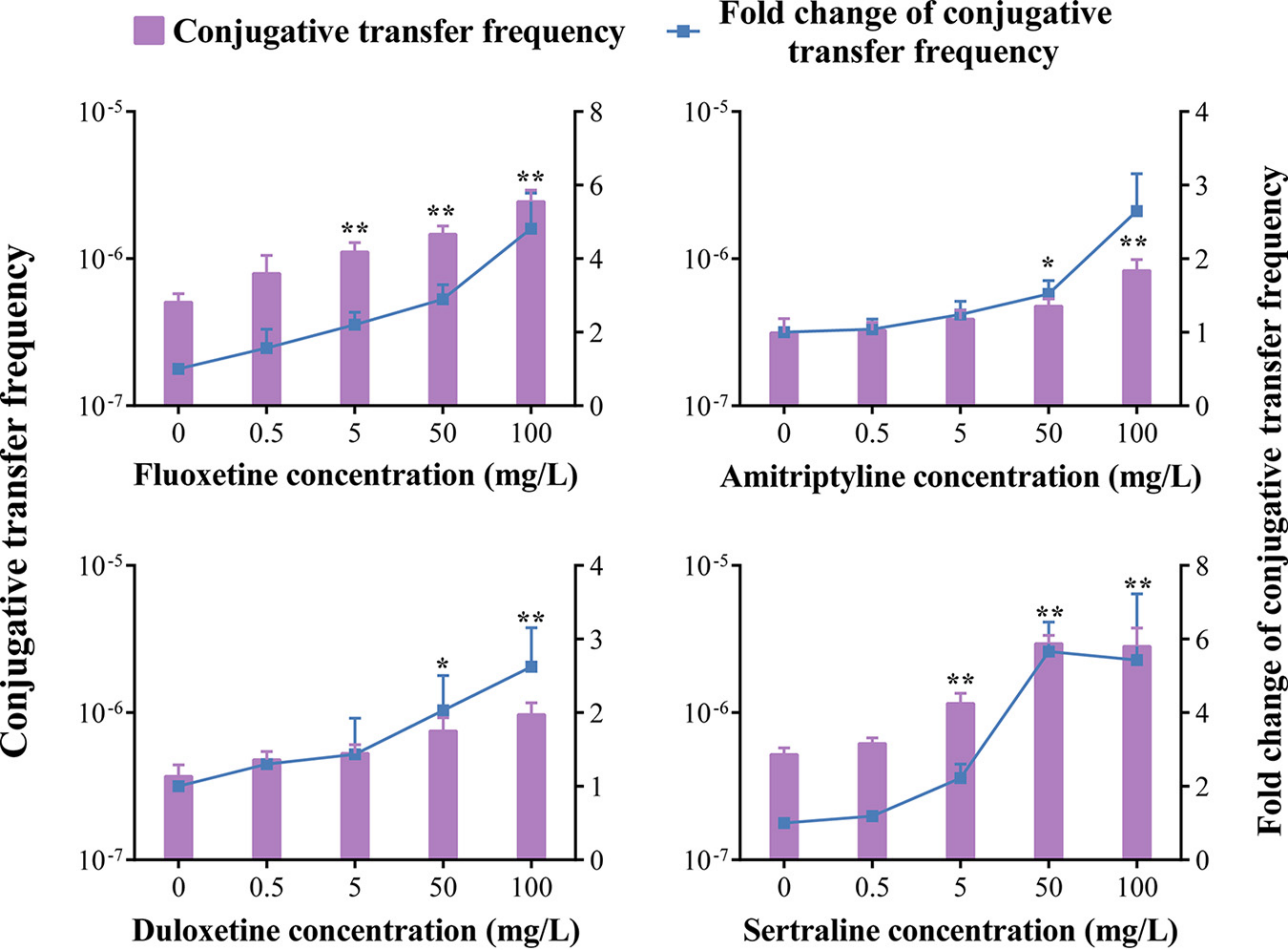
Effects of drug consumption on the frequency of conjugative transfer between E. coli HB101 and Salmonella Aberdeen after exposure to SGF at pH 4.5 for 4 h. The bars denote means ± SDs from three separate experiments. Significant differences are shown with * (*P < *0.05 versus the control [0 mg/L]) and ** (*P < *0.01 versus the control [0 mg/L]).

Previous studies have revealed that nonantibiotic pharmaceuticals, including fluoxetine and duloxetine, could induce multidrug resistance in E. coli through mutagenesis (15, 16). To assess the effects of drug consumption on the horizontal transfer of ARGs in SGF, we used four antidepressants (fluoxetine, amitriptyline, duloxetine, and sertraline) and assessed the conjugative transfer of ARGs between E. coli HB101 and Salmonella Aberdeen in SGF at pH 4.5 (Fig. 2). The conjugative transfer frequency was significantly increased (*P < *0.05) upon exposure to 50 mg/L or 100 mg/L fluoxetine, amitriptyline, sertraline, or duloxetine compared with that in the control group without drug exposure. Importantly, sertraline exposure had the maximum effect, resulting in a 5.66-fold increase in the conjugative transfer frequency compared with that in the control group. According to clinical recommendations, the prescribed daily doses of fluoxetine, duloxetine, sertraline, and amitriptyline are 20 to 120 mg (17–21). As the volumes of gastric fluid are approximately 30 mL on an empty stomach and 500 mL on a full stomach (22), antidepressant concentrations of 40 to 4,000 mg/L can be achieved in the stomach. Thus, antidepressant consumption could promote the horizontal transfer of ARGs in SGF at pH 4.5.

### Effects of dietary factors on the conjugative transfer of ARGs.

**FIG 3:**
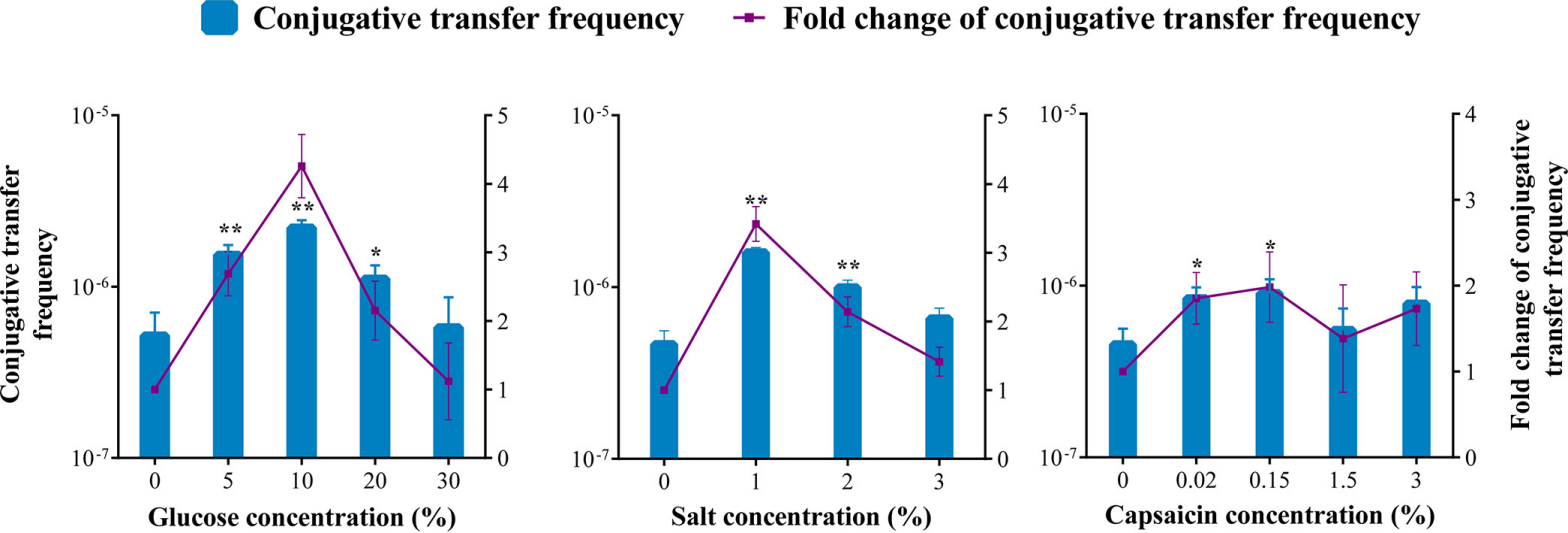
Effects of dietary factors on the frequency of conjugative transfer between E. coli HB101 and Salmonella Aberdeen after exposure to SGF at pH 4.5 for 4 h. The bars denote means ± SDs from three separate experiments. Significant differences are shown with * (*P < *0.05) and ** (*P < *0.01).

To assess the effects of certain dietary factors on the horizontal transfer of ARGs in SGF, conjugative transfer between E. coli HB101 and Salmonella Aberdeen was performed by adding seasonings to SGF at pH 4.5 (Fig. 3). Compared with the control group without any additions, the conjugative transfer frequency was significantly increased by 2.00- to 4.26-fold when the glucose concentration ranged from 5% to 20%, the salt concentration ranged from 1% to 2%, and the capsaicin concentration ranged from 0.02% to 3%. Notably, 10% glucose had the maximum effect, resulting in a 4.26-fold-higher conjugative transfer frequency than that in the control group. At present, a ≤5% daily value of added sugars per serving is considered low and a ≥20% daily value of added sugars per serving is considered high (23). The sugar contents of high- and medium-sugar soft drinks in the United Kingdom were >8 g/100 mL and 5 to 8 g/100 mL, respectively (24). Therefore, the sugar concentration in the stomach could reach 5% or higher if 100 mL of a soft drink was consumed per serving. According to the World Hypertension League, the recommended level of dietary salt intake is <5 g, with ≥5 g being considered high and >10 to 15 g being considered very high (25), resulting in very high salt concentrations of >1% per serving on a full stomach. The level of capsaicin consumption in Koreans can be up to 20 to 30 mg per day (26), reaching 0.03% per serving on an empty stomach. Thus, dietary factors such as high and medium sugar, very high salt, and high capsaicin concentrations could enhance the horizontal transfer of ARGs in SGF at pH 4.5.

### Mechanisms underlying conjugation promotion in SGF at pH 4.5.

**FIG 4:**
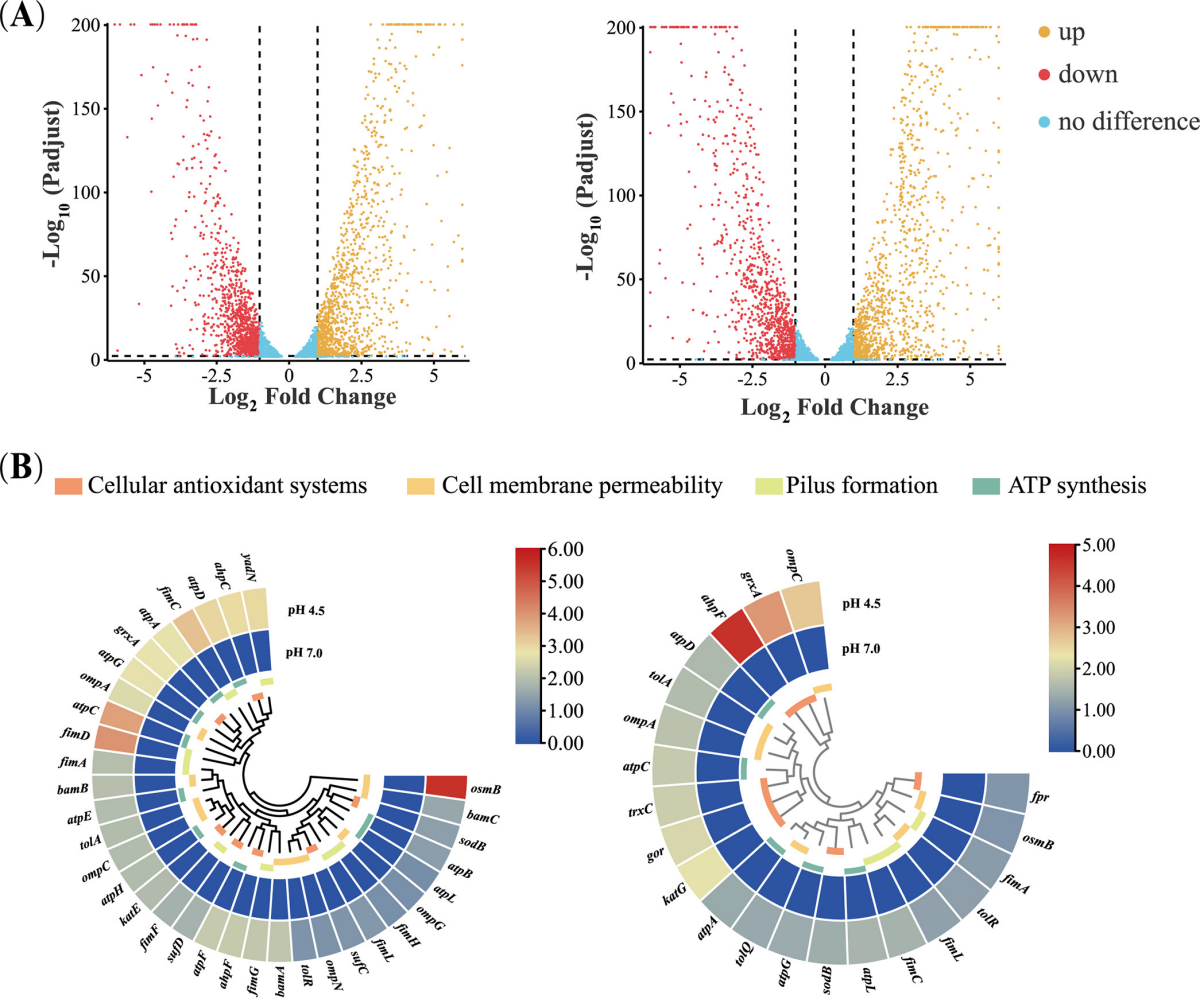
Transcriptomic analysis of DEGs in E. coli HB101 (left) and Salmonella Aberdeen (right) between the SGF group exposed at pH 4.5 and the control group exposed at pH 7.0 for 4 h. (A) Volcano map of DEGs. Padjust, adjusted *P* value. (B) Heat map of DEGs related to cellular antioxidant systems, cell membrane permeability, pilus formation, and ATP synthesis.

To explore the mechanisms underlying conjugation promotion in SGF at pH 4.5, we assessed the transcriptomic responses in donor E. coli HB101(RP4) cells and recipient Salmonella Aberdeen cells exposed to SGF at pH 4.5. We then validated our findings by measuring ROS levels, assessing cell membrane permeability, and performing RT-qPCR analysis. Compared with the control group at pH 7.0, totals of 1,647 genes were differentially upregulated and 1,660 genes were differentially downregulated in the donor E. coli HB101 cells, while 1,436 genes were differentially upregulated and 1,515 genes were differentially downregulated in the recipient Salmonella Aberdeen cells in the SGF group at pH 4.5 (Fig. 4A). These genes were involved in cellular antioxidant systems, cell membrane permeability, adhesive pilus formation, and ATP synthesis.

### (i) SGF at pH 4.5 induced ROS production and activated cellular antioxidant systems.

In general, ROS play an important role in the conjugation process (27). Low pH is an environmental stress factor that usually causes a series of physiological damages to bacterial cells (28), which may activate oxidant systems in these cells.

To study oxidative stress in bacteria, the expression levels of cellular antioxidant-encoding genes were assessed after exposure to SGF (Fig. 4B and Table S1). In the donor E. coli HB101 cells, exposure to SGF initiated a series of antioxidant defense systems. The expression levels of *ahpC*, *ahpF*, and *katE*, encoding the main enzymes that scavenge excessive hydrogen peroxide (29), were upregulated by 3.76- to 7.67-fold (*P < *0.01). Moreover, the expression level of *sodB*, the primary scavenger of O_2_^−^ (30), was upregulated by 2.66-fold (*P < *0.01). The expression level of *grxA*, which encodes glutaredoxins (31), was upregulated by 6.68-fold (*P < *0.01). The expression levels of other oxidative stress response genes, such as the Fe-S metabolism- and Fe-S cluster scaffold complex subunit-related genes *sufC* and *sufD*, were also upregulated by 2.35- to 3.34-fold in the donor cells. Similarly, the expression levels of *ahpF*, *grxA*, *katG*, and *sodB* were upregulated by 2.60- to 24.93-fold in the recipient cells (*P < *0.01). Genes encoding other antioxidant reductases, such as *gor*, *trxC*, and *fpr*, were also upregulated by 2.06- to 4.17-fold (*P < *0.01).

**FIG 5:**
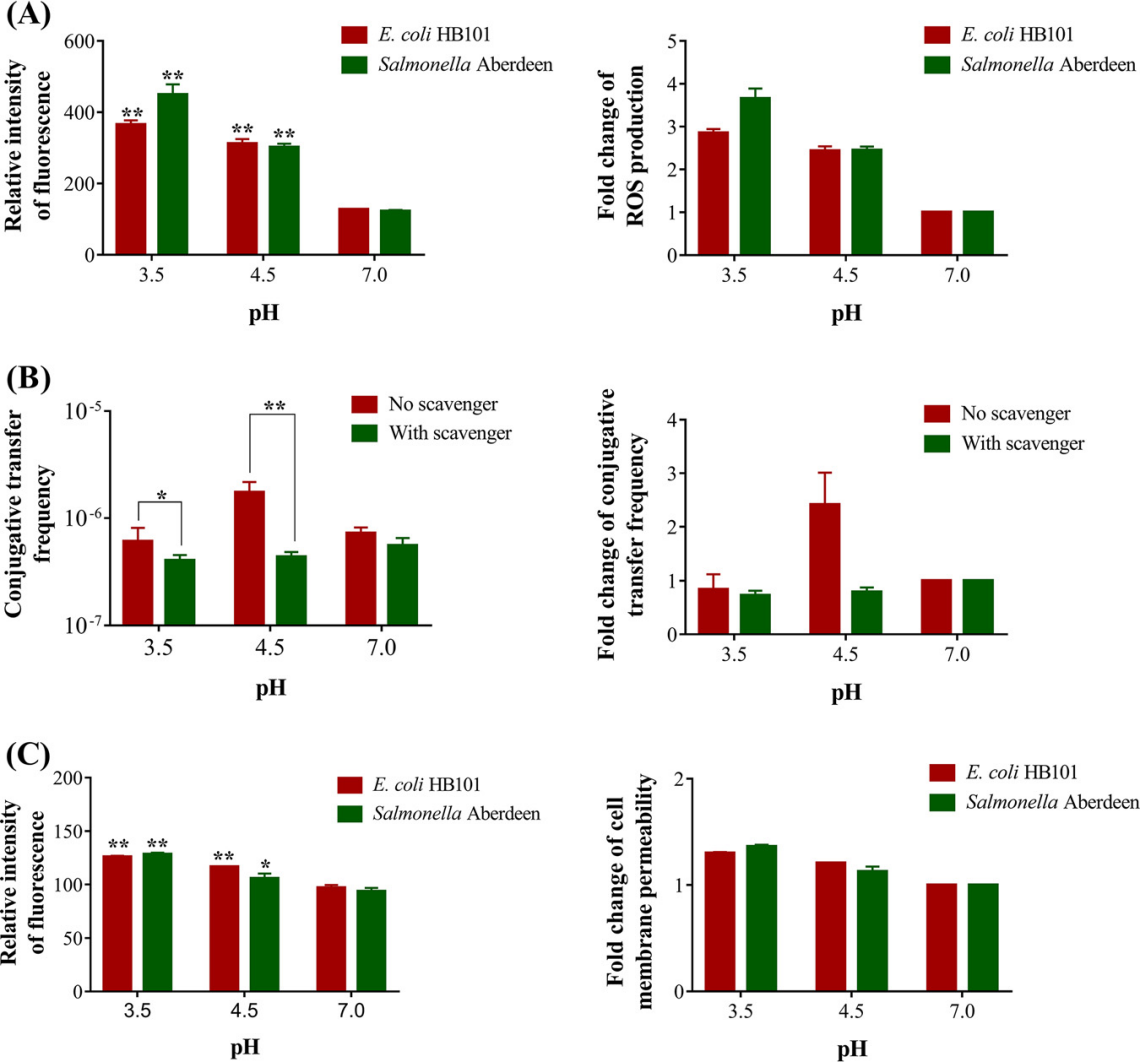
Effects of ROS production and cell membrane permeability on conjugative transfer. (A) Comparison of ROS levels (left) and fold changes in ROS production (right) between the SGF group exposed at pH 3.5 and 4.5 and the control group exposed at pH 7.0 for 4 h. (B) Comparison of conjugative transfer frequencies (left) and fold changes in the conjugative transfer frequencies (right) when an ROS scavenger was used versus when it was not used. (C) Comparison of cell membrane permeability (left) and fold changes in cell membrane permeability (right) between the SGF group exposed at pH 3.5 and 4.5 and the control group exposed at pH 7.0 for 4 h. The bars denote means ± SDs from three separate experiments. Significant differences are shown with * (*P < *0.05) and ** (*P < *0.01).

To validate the above-mentioned responses of cellular antioxidant systems, intracellular ROS levels were assessed for estimating the oxidation levels in bacterial cells. As shown in Fig. 5A, after exposure to SGF at pH 3.5 and 4.5 for 4 h, the ROS levels were significantly increased by 2.43- to 2.85-fold in the donor E. coli HB101 cells and by 2.45- to 3.66-fold in the recipient Salmonella Aberdeen cells (*P <* 0.01). Furthermore, ROS scavenging was performed to validate whether ROS production could enhance conjugative transfer upon exposure to SGF at pH 3.5 and 4.5. The frequency of the transfer of the RP4 plasmid across genera was significantly decreased by 1.50- to 3.98-fold upon exposure to SGF at pH 3.5 and 4.5, and there was no remarkable difference compared with the findings for the control group at pH 7.0 (*P < *0.01) (Fig. 5B). These results suggest that SGF-induced ROS overproduction plays a key role in enhancing the conjugative transfer of ARGs between bacteria.

### (ii) SGF at pH 4.5 increased cell membrane permeability.

Gram-negative bacteria possess various outer membrane proteins that are involved in conjugative transfer. In these bacteria, membrane permeability is modulated to facilitate communication between the donor and recipient cells by regulating the expression of genes encoding outer membrane porins, including *ompA*, *ompC*, and *ompF* (32, 33). Triclosan can increase the frequency of the conjugative transfer of ARGs by increasing cell membrane permeability (34).

Figure 4B presents the transcription levels of cell membrane-related genes in the donor and recipient cells after exposure to SGF at pH 4.5 (details are provided in Table S2 in the supplemental material). The expression levels of the porin-encoding genes *ompA*, *ompC*, *ompN*, and *ompG* were upregulated by 2.19- to 5.86-fold in the donor E. coli cells (*P < *0.01), and those of *ompA* and *ompC* were upregulated by 3.27- to 6.45-fold in the recipient Salmonella Aberdeen cells (*P < *0.01). Moreover, in the donor bacteria, the expression levels of outer membrane-related genes such as *bamA*, *bamB*, and *bamC*, which encode outer membrane protein assembly factors, were upregulated by 2.95- to 4.41-fold (*P < *0.01). Furthermore, 2.04- to 47.18-fold increases in the expression levels of *osmB*, encoding an outer membrane lipoprotein, and *tolA* and *tolR*, encoding proteins that maintain outer membrane stability, were noted in the donor and recipient cells (35). Further investigation of propidium iodide (PI)-positive cells revealed a significant increase by 1.12- to 1.37-fold at pH 3.5 and 4.5 compared with the findings for the control group (*P < *0.01) (Fig. 5C), indicating that exposure to SGF increased cell membrane permeability in the donor and recipient strains. Thus, an increase in membrane permeability could promote the occurrence of conjugative transfer.

### (iii) SGF at pH 4.5 promoted pilus formation.

**FIG 6:**
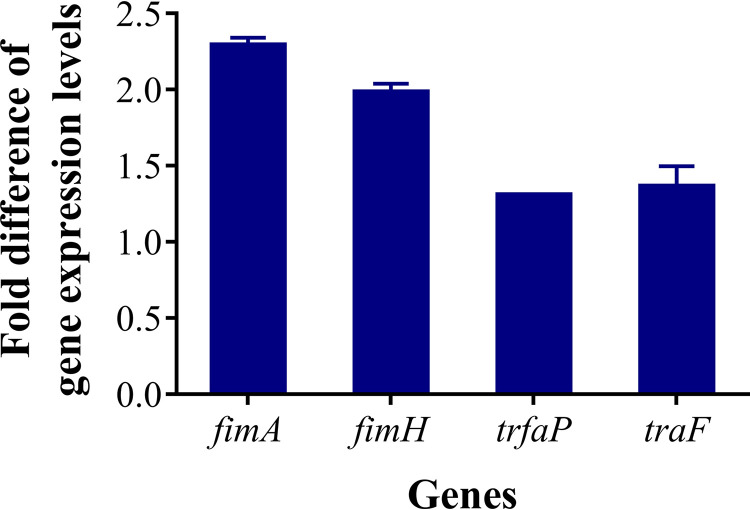
Differential expression of genes related to pilus formation and conjugative transfer upon exposure to SGF at pH 4.5. Relative concentrations of interesting genes were assessed by RT-qPCR. Fold differences in the expression levels of interesting genes between the SGF group exposed at pH 4.5 and the control group exposed at pH 7.0 were calculated. The primer sequences used are listed in Table S5 in the supplemental material. The bars denote means ± SDs from three separate experiments.

Direct cell-to-cell contact is necessary for plasmid transfer (36), and some *fim*-like genes have been characterized to contribute to cell adhesion in E. coli (37). Transcriptomic analysis revealed that the transcription levels of adhesion-related genes, including *fimD*, *fimC*, *fimA*, *fimL fimG*, *fimH*, *fimF*, and *yadN*, in the donor cells were significantly upregulated by 2.19- to 16-fold upon exposure to SGF at pH 4.5 (*P < *0.01) (Fig. 4B and Table S3). Similarly, the expression levels of *fimC*, *fimL*, and *fimA* were upregulated by 2.19- to 2.75-fold in the recipient cells (*P < *0.01). As shown in Fig. 6, qPCR analysis revealed that the relative expression levels of *fimA* and *fimH* in both bacteria were upregulated by 2.34-fold and 1.98-fold, respectively, upon exposure to SGF at pH 4.5. Thus, adhesive pili may play a crucial role in promoting conjugative transfer.

### (iv) SGF at pH 4.5 enhanced the expression levels of conjugative genes and promoted ATP synthesis.

The RP4 plasmid undergoes replication, partitioning, and conjugation during conjugative transfer. To assess the occurrence of conjugation upon exposure to SGF at pH 4.5, the transcription levels of genes related to plasmid transfer, namely, those related to the replication (Dtr) system initiated by promoter *trfap* and the mating pair formation system (*traF*) (38), were monitored by qPCR. The expression levels of these genes were upregulated by 1.35-fold and 1.31-fold, respectively (Fig. 6). In addition, DNA transfer during the conjugation process consumes energy, and increased energy availability can help improve conjugation (39). Differentially upregulated ATP-encoding genes that regulate cellular energy production are listed in Table S4. Notably, the transcription levels of *atpC*, *atpG*, *atpH*, *atpF*, *atpL*, *atpD*, *atpA*, *atpB*, and *atpE* in E. coli were upregulated by 2.83- to 24.08-fold (*P < *0.01), and those of *atpC*, *atpD*, *atpL*, *atpG*, and *atpA* in Salmonella Aberdeen were upregulated by 2.50- to 4.32-fold (*P < *0.01) (Fig. 4B and Table S4). Thus, enhanced expression levels of conjugative genes and increased ATP synthesis may indicate the acceleration of RP4 plasmid replication and transfer.

## DISCUSSION

At present, conjugation-mediated HGT is considered an important factor contributing to the antimicrobial resistance pandemic. The overuse and misuse of antibiotics have been identified as major factors contributing to the dissemination of antibiotic resistance, and most current studies are focusing on factors that influence conjugative transfer *in vitro*, e.g., disinfectants, nanoparticles, and heavy metals (40–42). However, so far, no study has assessed the effects of SGF and dietary factors on conjugative transfer.

The present study is the first to reveal that SGF at pH 3.5 or 4.5 can promote the horizontal transfer of ARGs between bacteria. Moreover, antidepressants such as fluoxetine, sertraline, duloxetine, and amitriptyline in SGF can enhance this transfer. In general, a gastric fluid pH of 1.0 to 2.0 is deleterious to many microbial pathogens in the stomach (43). However, the pH of gastric fluid can increase under certain circumstances, including malnutrition, the use of certain drugs, and various diseases (such as pernicious anemia) (44, 45). A typical American diet can increase the pH of the stomach to 4.0 to 5.0 (8). We found that the horizontal transfer of ARGs in the stomach may be increased under these circumstances, resulting in the dissemination of antibiotic resistance among the members of the bacterial community in the gastrointestinal tract. In other words, some diseases or an improper diet may reduce gastric acid levels and increase the risk of ARG transmission. Certainly, the cell numbers in the colon are higher than those in the stomach by ≥10^7^-fold (46), resulting in the number of transconjugants arising in the colon also being ≥10^7^-fold higher. Therefore, an increase in the number of transconjugants in the stomach may be minimal compared to the orders-of-magnitude difference in the colon.

To assess why SGF could promote the conjugative transfer of ARGs at pH 4.5, we performed a transcriptomic analysis. Previously, increased ROS production or membrane permeability due to CuO nanoparticles and Cu^2+^ was found to be a common cause of the conjugative transfer of the RP4 plasmid (47). Accordingly, in our study, the donor E. coli cells and recipient Salmonella Aberdeen cells exposed to SGF at pH 4.5 exhibited elevated levels of ROS and increased expression levels of genes related to antioxidative stress, such as *ahpF* and *katE*, indicating that the strains overexpressed antioxidant proteins as a defense mechanism to protect themselves from acid-induced ROS stress. Furthermore, cell membrane permeability-related genes (e.g., *ompA* and *ompC*, which encode outer membrane proteins) were upregulated, suggesting that membrane permeability could be increased and contribute to the increased conjugative transfer of ARGs in SGF at pH 4.5. To enable the transfer of plasmid DNA from donor to recipient bacteria, physical cell-to-cell contact is necessary during the conjugation process. Type 1 pili have been shown to increase the adhesion of donor E. coli K-12 cells to neighboring cells (48). In our study, most adhesion-related genes (e.g., *fim*) were expressed in the donor and recipient cells at pH 4.5, indicating that adhesive pili may enhance plasmid transfer by facilitating cell-to-cell contact between donors and recipients. Thus, SGF at pH 4.5 could increase the conjugative transfer of the RP4 plasmid through ROS overproduction and could enhance cell membrane permeability and adhesive pilus formation.

In summary, we found that SGF at pH 3.5 and 4.5 could enhance the conjugative transfer of ARGs within and across bacterial genera. Furthermore, the consumption of antidepressants, including amitriptyline, fluoxetine, duloxetine, and sertraline, and certain dietary factors, including very high salt, high and medium sugar, and high capsaicin concentrations, could facilitate conjugative transfer. The induction of ROS production, the activation of cellular antioxidant systems, increases in cell membrane permeability, and the promotion of adhesive pilus formation were factors potentially contributing to an increase in the transfer efficiency in SGF at elevated pH levels. These results broaden our understanding of ARG transmission in the gastrointestinal tract and reveal that some diseases or improper dietary intake may decrease gastric acid levels and increase the risk of ARG dissemination.

## MATERIALS AND METHODS

### Antibiotics, bacterial strains, and culture conditions.

Antibiotics (ampicillin [Amp], kanamycin [Kan], tetracycline [Tet], chloramphenicol [Chl], and streptomycin [Str]) were purchased from Sigma-Aldrich. E. coli HB101 containing the RP4 plasmid (60,099 bp) and exhibiting Amp, Kan, and Tet resistance was chosen as the donor. E. coli K-12 exhibiting Chl resistance, obtained from our previous mutation study (16), and Salmonella Aberdeen exhibiting Str resistance, purchased from the American Type Culture Collection (ATCC 50312), were chosen as the recipients. E. coli HB101 was cultured in Luria-Bertani (LB) medium (Oxoid, UK) containing 100 mg/L Amp, 60 mg/L Kan, and 40 mg/L Tet. E. coli K-12 was cultured in LB medium containing 25 mg/L Chl, and Salmonella Aberdeen was cultured in LB medium containing 25 mg/L Str. All of the strains were incubated at 37°C for 16 h.

### Preparation of SGF.

SGF was prepared according to a previously described method (49). In brief, 1 L of SGF contained 8.3 g of proteose peptone, 3.5 g of d-glucose, 2.05 g of NaCl, 0.6 g of KH_2_PO_4_, 0.37 g of KCl, and 0.11 g of CaCl_2_. The SGF solution was autoclaved at 121°C for 15 min. After the pH was adjusted using 5.0 N HCl, 5 mL of a solution of 2.66 mg/mL of pepsin, 20 mg/mL of lysozyme, and 10 mg/mL of ox bile was sterilized using a filter with a pore size of 0.22 μm and added to 1 L of SGF.

### Conjugation experiments.

Donor E. coli HB101(RP4) and recipient E. coli K-12 and Salmonella Aberdeen cells were freshly grown in LB medium at 37°C for 14 to 16 h. Next, the cells were centrifuged at 8,000 × *g* for 5 min. After the removal of the supernatants, the pellets were washed three times with phosphate-buffered saline (PBS) (pH 7.0) and resuspended in PBS to obtain a cell count of ~10^9^ CFU/mL. Following this, 100 μL of the donor cells and 100 μL of the recipient cells were added to 8 mL of the SGF solution at different pH levels (1.5, 2.5, 3.5, or 4.5) or PBS (pH 7.0) for 2 and 4 h to achieve a final concentration of ~10^8^ CFU/mL. Subsequently, 500 μL of the bacterial mixtures was plated onto LB agar plates and incubated at 37°C for 36 h. For transfer within genera, transconjugants were selected on LB agar plates containing 100 mg/L Amp, 40 mg/L Tet, 60 mg/L Kan, and 25 mg/L Chl. The recipient bacterial count was determined by plating diluted mating mixtures onto LB agar plates containing 25 mg/L Chl. For transfer across genera, transconjugants were selected on LB agar plates containing 100 mg/L Amp, 40 mg/L Tet, 60 mg/L Kan, and 25 mg/L Str. The recipient bacterial count was determined by pouring the diluted mating mixtures onto LB agar plates containing 25 mg/L Str. The conjugation frequency was calculated by dividing the number of verified transconjugant colonies by the total number of recipients. All of the experiments were performed in at least biological triplicates.

To assess the effects of drug consumption, dietary factors, and ROS scavenging on the conjugative transfer of the RP4 plasmid, four antidepressants (fluoxetine, amitriptyline, duloxetine, and sertraline at 0.5 to 100 mg/L), three dietary factors (5% to 30% glucose, 1% to 3% salt, and 0.02% to 3% capsaicin), and 100 μmol/L of thiourea were added to SGF at pH 4.5, and the frequency of conjugation between E. coli HB101 and Salmonella Aberdeen was assessed.

### Verification of transconjugants.

To verify the formation of transconjugants carrying the RP4 plasmid, transconjugant E. coli K-12 and Salmonella Aberdeen cells were randomly selected and cultured in LB broth containing 100 mg/L Amp, 40 mg/L Tet, 60 mg/L Kan, and 25 mg/L Chl or 25 mg/L Str at 37°C for 14 to 16 h. The plasmid was extracted using a plasmid extraction kit (Tiangen, China) according to the manufacturer’s instructions and assessed using 1% agarose gel electrophoresis. The unique gene *traG* in the RP4 plasmid was selected as the target gene and verified by PCR. The RP4-specific primers used for PCR were RP4-f (5′-AAAGCGGACAGCATCAGTAACGAA-3′) and RP4-r (5′-GAGCTTGGTGGCCGCATAGTGTAG-3′). The reaction was performed using a 20-μL reaction mixture containing 10 μL of 2× Rapid *Taq* master (TaKaRa Bio Inc., Japan), 1 μL of each primer (10 μmol/L), 2 μL of the extracted plasmids, and 6 μL of nuclease-free water. The thermal cycling conditions were as follows: an initial denaturation step at 95°C for 5 min followed by 30 cycles at 95°C for 30 s, 55°C for 30 s, and 72°C for 1 min and a final extension step at 72°C for 5 min. The PCR product was 104 bp and was assessed using 1% agarose gel electrophoresis (see Fig. S1 in the supplemental material).

### Measurement of ROS levels.

ROS production was assessed by flow cytometry (Bio-Rad, USA) using the 2′,7′-dichlorofluorescein diacetate (DCF-DA) cellular ROS detection assay kit (Nanjing Jiancheng Bioengineering Institute, China), according to the manufacturer’s instructions. In brief, suspensions of ~10^6^ CFU/mL of donor and recipient cells in PBS were incubated with DCF-DA for 30 min at 37°C. Next, the DCF-DA-loaded cells were treated with SGF at different pH levels (3.5 or 4.5) or PBS (pH 7.0) for 30 min. All of the samples were scanned by flow cytometry at an excitation wavelength of 488 nm and an emission wavelength of 526 nm. All of the tests were performed in biological triplicates.

### Assessment of cell membrane permeability.

Cell membrane permeability was assessed by flow cytometry, as described previously (34). In brief, after the donor and recipient bacterial cells were treated with SGF at different pH levels (3.5 or 4.5) or PBS (pH 7.0) for 30 min, propidium iodide (PI) (final concentration of 30 μM) was added to the cells, and the cells were incubated for 30 min at 30°C in the dark. All of the samples were scanned by flow cytometry at an excitation wavelength of 488 nm and an emission wavelength of 630 nm. All of the experiments were performed in at least triplicates.

### RNA extraction and transcriptome resequencing.

Donor E. coli HB101(RP4) and recipient Salmonella Aberdeen cells were prepared as described above. Next, 100 μL of the donor cells and 100 μL of the recipient cells were added to an 8-mL SGF solution at pH 4.5. After mating for 4 h across genera, the bacterial cells were collected by centrifugation at 8,000 × *g* for 10 min. Total RNA was extracted using the RNeasy minikit (Qiagen, Germany), according to the manufacturer’s instructions. Sequencing was performed by Novogene (Beijing, China). In the control group, 100 μL of the donor cells and 100 μL of the recipient cells were added to 8 mL of PBS at pH 7.0. Global transcriptional analysis was performed according to methods described in a previous study, with a few modifications (50). Quality control was applied to raw sequencing reads before they were mapped against the genomes of E. coli HB101 (GenBank accession number NC_000913.3) and Salmonella Aberdeen (accession number NC_003197.2). Cufflinks (version 2.2.1) was used to analyze differential expression in triplicate cultures under the same conditions. The fragments per kilobase of transcript per million mapped reads (FPKM) method of gene expression analysis was used to identify differentially expressed genes (DEGs) between the SGF group at pH 4.5 and the control group at pH 7.0. The resultant *P* values were adjusted using the Benjamini-Hochberg approach (51) for controlling the false discovery rate. A stringent absolute log_2_ fold change cutoff of ≥1 with an adjusted *P* value of <0.05 was adopted to distinguish the DEGs.

### Quantification of gene expression by RT-qPCR.

The extracted RNA was transcribed to cDNA by RT-PCR using a reverse transcription kit (TaKaRa Bio Inc., Japan). Next, qPCRs were performed using 20 μL of a reaction mixture consisting of 2 μL of cDNA, 10 μL of SYBR green *Taq*, 0.5 μL of each primer (10 μmol/L), and 7 μL of RNase-free water. The PCR conditions were as follows: an initial step at 95°C for 10 min, 40 cycles at 95°C for 15 s and 60°C for 1 min, and a final step at 95°C for 15 s and 60°C for 1 min. All qPCRs were performed in triplicates using diethyl pyrocarbonate (DEPC)-treated water as the negative control. qPCR was performed using the ABIViiA 7 real-time PCR system (Applied Biosystems, USA). All of the primers are listed in Table S5.

After determining the threshold cycle (*C_T_*) values for both the reference (16S rRNA) and target genes, the relative expression levels of target mRNAs were calculated by normalization against 16S rRNA gene expression levels. The control group at pH 7.0 was used for calibration. A comparative ΔΔ*C_T_* method (2^−ΔΔ^*^CT^*) was used to assess gene expression differences from triplicate measurements (52).

### Statistical analyses.

GraphPad Prism 7.00 (GraphPad Inc., San Diego, CA, USA) and the TBtools (version 1.108) data visualization tool were used for graphic drawing. The SPSS Statistics 22.0 software package (IBM Corp., Armonk, NY, USA) was used for all statistical analyses. Significant differences were assessed using the independent-sample *t* test. *P* values of <0.05 were considered significant, and *P* values of <0.01 were considered highly significant.

### Data availability.

RNA sequencing data are accessible through the Sequence Read Archive (SRA) under BioProject accession number PRJNA930988.
