# Supplementary material for: Simulated Gastric Acid Promotes the Horizontal Transfer of Multidrug Resistance Genes across Bacteria in the Gastrointestinal Tract at Elevated pH Levels
Source: Microbiol Spectr. 2023 Apr 18;11(3):e04820-22. doi: 10.1128/spectrum.04820-22 (PMC10269839; doi:10.1128/spectrum.04820-22)
Supplement: Supplemental file 1 — Supplemental material. Download spectrum.04820-22-s0001.pdf, PDF file, 0.4 MB [file spectrum.04820-22-s0001.pdf]

Supplement Materials

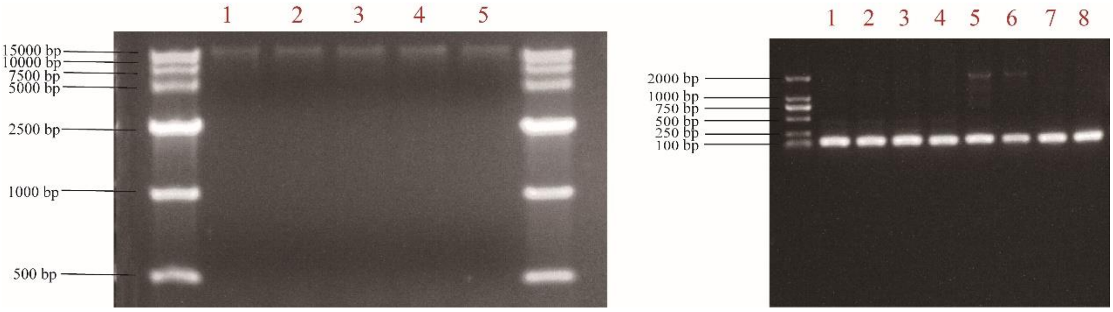

Fig. S1. Electrophoresis of the RP4 plasmids and PCR products of *traG* gene. (A) RP4 plasmids, panel 1-5; (B) *traG* gene, panel 1-8.

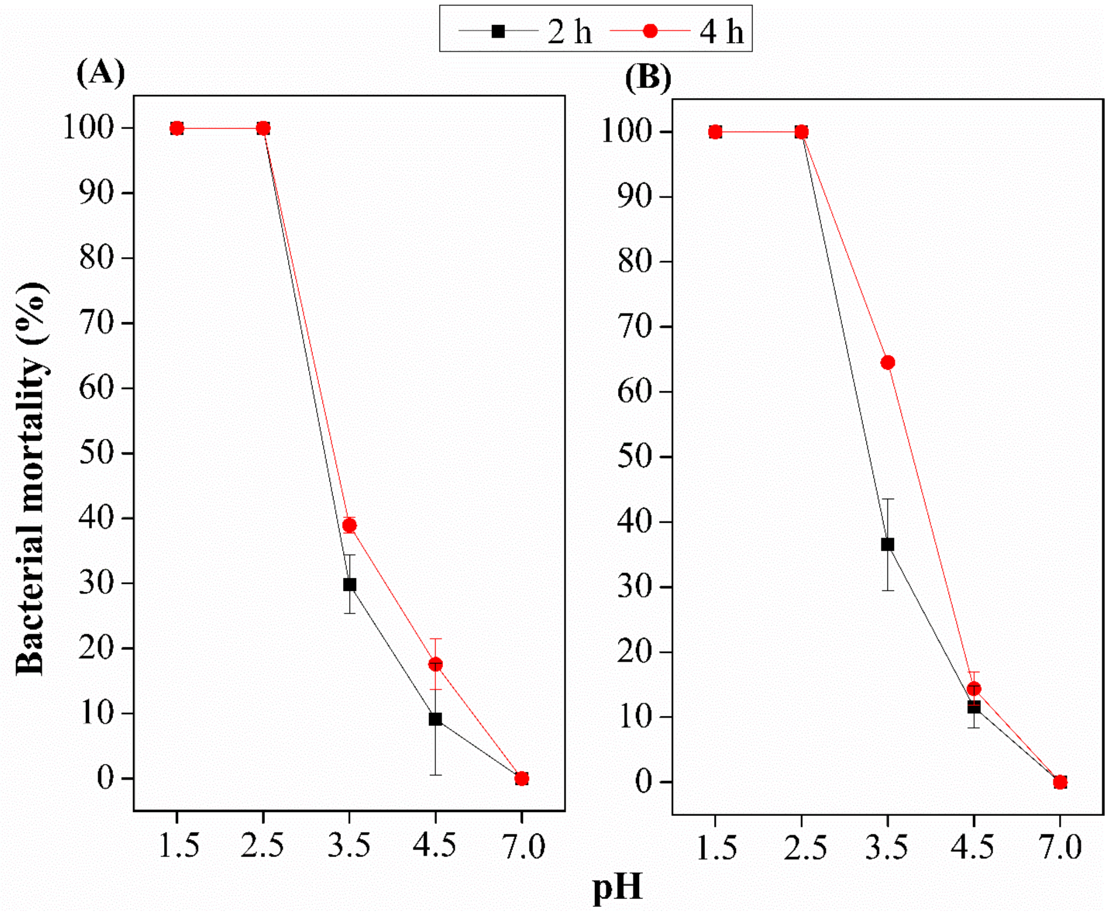

Fig. S2. Effect of SGF pH and exposure time on the bacterial mortality of the donor *E. coli* HB101 (A) and recipient *Salmonella* Aberdeen (B).

Table S1. Changes in the expression of genes related to oxidative stress in *E. coli* HB101 and *Salmonella* Aberdeen after exposure to SGF at pH4.5.

Strain	Gene ID	Genes	Fold change	Function
E. coli	b0605	*ahpC*	7.67	alkyl hydroperoxide reductase 2C
	b0606	*ahpF*	4.69	alkyl hydroperoxide reductase 2C
	b0849	*grxA*	6.68	reduced glutaredoxin 1
	b1681	*sufD*	3.34	Fe-S cluster scaffold complex subunit
	b1682	*sufC*	2.35	Fe-S cluster scaffold complex subunit
	b1656	*sodB*	2.66	superoxide dismutase
	b1732	*katE*	3.76	katE catalase HPII
Salmonella	STM0609	*ahpF*	24.93	pyridine nucleotide-disulphide oxidoreductase
	STM0872	*grxA*	9.85	reduced glutaredoxin 1
	STM4106	*katG*	4.82	peroxidase
	STM1431	*sodB*	2.60	superoxide dismutase
	STM3597	*gor*	4.17	pyridine nucleotide-disulphide oxidoreductase
	STM2649	*trxC*	3.78	thioredoxin
	STM4084	*fpr*	2.06	pxidoreductase NAD-binding domain

Table S2. Changes in the expression of genes related to cell membrane in *E. coli* HB101 and *Salmonella* Aberdeen after exposure to SGF at pH4.5.

Strain	Gene ID	Genes	Fold change	Function
E. coli	b0957	*ompA*	5.86	outer membrane protein A
	b2215	*ompC*	3.81	outer membrane porin C
	b1377	*ompN*	2.28	outer membrane porin N
	b1319	*ompG*	2.19	outer membrane porin G
	b0177	*bamA*	4.41	outer membrane protein assembly factor
	b2512	*bamB*	3.97	outer membrane protein assembly factor
	b2477	*bamC*	2.95	outer membrane protein assembly factor
	b1283	*osmB*	47.18	outer membrane lipoprotein
	b0739	*tolA*	3.81	Tol-Pal system protein
	b0738	*tolR*	2.30	Tol-Pal system protein
Salmonella	STM2267	*ompC*	6.45	outer membrane porin C
	STM1070	*ompA*	3.27	outer membrane protein A
	STM0747	*tolA*	3.05	Tol-Pal system protein
	STM0745	*tolQ*	2.48	Tol-Pal system protein
	STM0746	*tolR*	2.28	Tol-Pal system protein
	STM1705	*osmB*	2.04	outer membrane lipoprotein

Table S3. Changes in the expression of genes related to pilus formation in *E. coli* HB101 and *Salmonella* Aberdeen after exposure to SGF at pH4.5.

Strain	Gene ID	Genes	Fold change	Function
E. coli	b4317	*fimD*	16.00	type I fimbriae usher protein
	b4318	*fimC*	10.06	type 1 fimbriae minor subunit
	b4314	*fimA*	3.94	type 1 fimbriae major subunit
	b4315	*fimL*	2.45	putative fimbriae protein
	b4319	*fimG*	4.44	type 1 fimbriae minor subunit
	b4320	*fimH*	2.19	type 1 fimbriae D-mannose specific adhesin
	b4318	*fimF*	3.29	type 1 fimbriae minor subunit
	b0141	*yadN*	7.84	putative fimbrial protein
Salmonella	STM0545	*fimC*	2.75	pili assembly chaperone protein
	STM0544	*fimL*	2.33	fimbrial protein
	STM0543	*fimA*	2.19	fimbrial protein

Table S4. Changes in the expression of genes related to ATP synthase *E. coli* HB101 and *Salmonella* Aberdeen after exposure to SGF at pH4.5.

Strain	Gene ID	Genes	Fold change	Function
E. coli	b3731	*atpC*	14.12	ATP synthase
	b3732	*atpD*	8.17	ATP synthase
	b3734	*atpA*	6.59	ATP synthase
	b3733	*atpG*	6.50	ATP synthase
	b3736	*atpF*	4.59	ATP synthase
	b3737	*atpE*	3.86	ATP synthase
	b3735	*atpH*	3.81	ATP synthase
	b3738	*atpB*	2.64	ATP synthase
	b3739	*atpL*	2.14	ATP synthase
Salmonella	STM3864	*atpC*	3.58	ATP synthase
	STM3865	*atpD*	2.95	ATP synthase
	STM3872	*atpL*	2.83	ATP synthase
	STM3866	*atpG*	2.53	ATP synthase
	STM3867	*atpA*	2.50	ATP synthase

Table S5. Primer sequences used in this study

Genes	Primer	Sequences (5'-3')	Length/bp	References
fimA	*fimA*-F	atctaaagccgctgttgcct	117	(1)
	fimA-R	tccaggatctgcacaccaac		
fimH	*fimH*-F	gtgccaattcctcttaccgtt	123	(1)
	fimH-R	tggaataatcgtaccgttgcg		
trfap	*trfAp-F*	gaagcccatcgccgtcgcctgtag	131	(2)
	trfAp-R	gccgacgatgacgaactggtgtgg		
traF	*traF-F*	ggcaacctcgtcgccttta	118	(2)
	traF-R	gcaagtcggcgtgttttcg		
16S rRNA	*16s-F*	cctacgggaggcagcag	194	(2)
	16s-R	attaccgcggctgctgg		

References

1. Zhang P, Mao D, Gao H, Zheng L, Chen Z, Gao Y, Duan Y, Guo J, Luo Y, Ren H. 2022. Colonization of gut microbiota by plasmid-carrying bacteria is facilitated by evolutionary adaptation to antibiotic treatment. ISME J 16:1284-1293. https://doi.org/10.1038/s41396-021-01171-x.

2. Zhang Y, Gu AZ, Cen T, Li X, Li D, Chen J. 2018. Petrol and diesel exhaust particles accelerate the horizontal transfer of plasmid-mediated antimicrobial resistance genes. Environ Int 114:280-287. https://doi.org/10.1016/j.envint.2018.02.038.
